# A12 STEPWISE COORDINATION OF COLONIC NEUTROPHILS AND INNATE LYMPHOID CELLS IN THE ONSET AND RESOLUTION OF CLOSTRIDIOIDES DIFFICILE TOXIN-INDUCED INJURY

**DOI:** 10.1093/jcag/gwab049.011

**Published:** 2022-02-21

**Authors:** K L Flannigan, A Serra, S A Hirota

**Affiliations:** Physiology & Pharmacology, University of Calgary, Calgary, AB, Canada

## Abstract

**Background:**

While our understanding and use of treatments for *Clostridioides difficile* infection (CDI) has improved, initial CDI still carries significant morbidity and mortality owing to heterogeneity in host immune responses. Further, host immunity is a critical modulator of fecal microbiota transplantation (FMT) success in CDI. Thus, understanding the host immune response during CDI is essential.

**Aims:**

To assess the cellular immune responses that trigger the onset and resolution of injury and inflammation in CDI.

**Methods:**

Colonic injury and inflammation triggered by CDI was modelled in mice using intrarectal installation of *C. difficile* toxins A and B (TcdA/B). Colonic tissue was collected at various timepoints following TcdA/B exposure to assess gene expression (qPCR), cytokine production (ELISA) and immune cell responses (flow cytometry). Knockout mice and neutralizing antibodies were used to deplete cytokines or cells.

**Results:**

Examinion of colonic gene expression at different times following TcdA/B exposure found a dominant transcriptional signature related to neutrophil adhesion and diapedesis. In addition to the typical neutrophil chemokines *Cxcl1* and *Cxcl2*, TcdA/B exposure also increased expression of neutrophil effector genes including *Elane* (neutrophil elastase). Neutrophil influx in response to TcdA/B was a critical driver of intestinal injury as antibody-mediated depletion of neutrophils lead to significantly less damage in the colon following TcdA/B exposure. Along with neutrophil influx, there were high levels of antimicrobial gene expression in the colon after TcdA/B exposure including *RegIIIγ*, *S100a8*, and *Socs3*, all genes regulated by IL-22. Upon further investigation, IL-22 was a significant mediator in the host response to TcdA/B exposure as it was upregulated >150-fold in the colon and originated from type 3 innate lymphoid cells (ILC3). Further, TcdA/B exposure in IL-22^-/-^ mice lead to significantly more colonic damage compared to wildtype (WT) mice. Subsequent screening of previously published RNAseq data from IL-22-treated mouse colonic organoids identified various upregulated proteins involved in immune regulation, including the gene *Slpi* that encodes a protein (secretory leukocyte peptidase inhibitor) that inhibits leukocyte proteases, including neutrophil elastase. While TcdA/B challenge robustly induced the expression of *Slpi* in the colon of WT mice, IL-22^-/-^ mice failed to express increased levels of *Slpi* and had greater levels of neutrophil elastase activity in the colon.

**Conclusions:**

Together these data suggest a stepwise immune response to TcdA/B where ILC3 produce IL-22 to induce epithelial release of SLPI that attenuates the damaging effects of early neutrophil responses. Strategies to upregulate IL-22 may help control damage triggered by CDI and promote resolution of injury.

**Funding Agencies:**

Lloyd Sutherland Chair in GI Research, Canadian Research Chair

